# Second Iteration of Photogrammetric Processing to Refine Image Orientation with Improved Tie-Points †

**DOI:** 10.3390/s18072150

**Published:** 2018-07-04

**Authors:** Nguyen Truong Giang, Jean-Michaël Muller, Ewelina Rupnik, Christian Thom, Marc Pierrot-Deseilligny

**Affiliations:** Laboratoire en Sciences et Technologies de l’information Géographique, Institut National de l’information Géographique et Forestière, Saint-Mandé, 94160, France; Truong-Giang.Nguyen@ign.fr (N.T.G.); Jean-Michael.Muller@ign.fr (J.-M.M.); Ewelina.Rupnik@ign.fr (E.R.); Christian.Thom@ign.fr (C.T.)

**Keywords:** tie-points, bundle block adjustment, image orientation, camera calibration, precision mapping

## Abstract

Photogrammetric processing is available in various software solutions and can easily deliver 3D pointclouds as accurate as 1 pixel. Certain applications, e.g., very accurate shape reconstruction in industrial metrology or change detection for deformation studies in geosciences, require results of enhanced accuracy. The tie-point extraction step is the opening in the photogrammetric processing chain and therefore plays a key role in the quality of the subsequent image orientation, camera calibration and 3D reconstruction. Improving its precision will have an impact on the obtained 3D. In this research work we describe a method which aims at enhancing the accuracy of image orientation by adding a second iteration photogrammetric processing. The result from the classical processing is used as *a priori* information to guide the extraction of refined tie-points of better photogrammetric quality. Evaluated on indoor and UAV acquisitions, the proposed methodology shows a significant improvement on the obtained 3D point accuracy.

## 1. Introduction

Products of photogrammetric processing are nowadays used across many domains, and are available via commercial and open-source software tools. The quality demands of such products vary with applications, e.g., from millimetric accuracy in industrial metrology [1] to centimetric or decentimetric accuracies in applications concerning indoor mapping [2], cultural heritage documentation [3], mobile mapping [4] or airborne surveys [5,6]. Typically, as the accuracies grow, so does the on-site labour to prepare an acquisition.

3D measurements derived from images are a product of several successive operations: image tie-point extraction, camera calibration and orientation, 3D triangulation. As they are all functionally related, the tie-point measurement precision will have an impact on the precision of the calculated calibrations, orientations and ultimately the triangulated 3D points. The importance of image observations on the camera modelling has been long known and proven empirically [7]. Poor precision and spatial distribution of the tie-points inhibits both: reliable estimation of the camera orientation and proper modelling of the systematic errors produced by the optics. As a consequence, output pointclouds become deformed and inaccurate [8,9]. To fight against this effect, a lot of research has been focused on utilizing only those tie-points that guarantee the so-called photogrammetric quality, i.e., (i) favour image points with low re-projection errors, (ii) keep good point distribution in image and object spaces, (iii) maintain reasonable intersection angles, and (iv) maximize points’ manifold [3,10,11,12].

In this research we address totally the above aspects in a novel processing framework that is capable of furnishing 3D accuracies improved by a factor of two with respect to the *state-of-the-art*. Starting from the result of the classical processing (i.e., tie-point extraction, camera calibration and orientation, dense image matching), the method automatically generates a new set of tie-points that maintains the optimal distribution, good manifold and enhanced measurement precision.

The publication is structured as follows. A concise review on *state-of-the-art* tie-point extraction approaches is presented below, followed by the mention of the research contributions. In Section 2 the detailed theoretical and algorithmic base is given. Finally, Section 3 describes the performed experiments and discuses the obtained results.

### 1.1. Related Works

The tie-points are the interest points (or keypoints) that had been extracted and subsequently matched with their correspondences. Interest points alone are salient features (e.g., points or regions) that are distinctive in their immediate neighbourhood. The performance of an interest point detector is evaluated in terms of repeatability and information content [13]. A good interest point detector is invariant to a class of transformations: translation, rotation, scale and perspective distortions. It is also insensitive to illumination change.

Over the course of the last twenty years many different interest operators have been invented that would implement these properties. The distinctiveness of a pixel can be measured by its local dissimilarity, e.g., with the auto-correlation function to determine the signal changes [14,15] (e.g., *Moravec*, *Harris*), with covariance matrices [16] (*Foerstner*), using comparison functions on circular masks [17,18] (e.g., *SUSAN*, *FAST*), or the spatial gradient distribution [19] (e.g., *Scale Invariant Feature Transform or SIFT*). To include scale invariance, the notion of 3D scale-space representation was introduced [20]. The images would be represented by a pyramid of, e.g., Laplacian-of-Gaussians (LoG) or difference-of-Gaussians (DoG), where the pyramid’s maxima were the characteristic points [19,21,22] (e.g., SIFT; *Speeded-up robust features or SURF*). Scale invariant point detectors, however, assume a uniform scale-change in both directions therefore do not model perspective (or affine) deformations induced by the imaging process. The end effect is that the aforementioned operators identify corresponding points between image couples with very small base-to-height ratios and fail at wide-baseline scenarios. To address this shortcoming, the known operators were affine-adapted and new affine-invariant operators were devised. Affine-invariant point detectors often delineate the so-called distinguished regions (DR) of stable properties and high repeatability. The DRs can be, for instance, parallelograms built on the Harris points and their nearby edges [23], elliptical blobs calculated from Harris-Laplace operator and the local structure described by the second moment matrix [21] (*Harris-Affine*, *Hessian-Affine*) or blobs of arbitrary shapes [24,25] (*Maximally Stable Extremal Regions or MSER*). Description of such regions is accomplished by identifying a local affine coordinate system or by geometric normalising, and establishing a description vector from the intensities within the region.

Out of the most popular affine-invariant region detectors, the MSER is said to be the most repeatable in the presence of changing viewpoint angles, scales, blur and illumination [26]. As proved experimentally [10], a notable point detector robust to changing viewpoints is the SIFT, and its affine adaption *affine-SIFT* (*ASIFT*) [27]. In the latter, the authors combine the concepts of simulation and normalization to simulate all distortions a patch can undergo when tilting the image. Next, they compare the totality of the generated images with SIFT. Computational time turns to be a restraining issue of the algorithm.

Regarding the measurement precision of the most popular tie-point extraction algorithms, the bundle block adjustement (BBA) reprojection errors place in the range of 0.5–1 pixel [10,11]. Higher precision (≈0.1 pixel) can be obtained by coupling interest point detectors with e.g., *Least Squares Matching* (LSM) [3,28,29].

### 1.2. Contributions

The aforementioned algorithms extract image tie-points without making any *a priori* on the camera or the geometry of the 3D scene. In this research work, our hypothesis is that the camera orientations and approximate 3D geometry is known, and then we exploit it to guide and calculate a new set of tie-points of improved quality. Accordingly, we call it the Second Iteration to Refine image Orientation with improved Tie-Point (*SIROP*). More precisely, contributions of this research work are as follows

**Figure 1:**
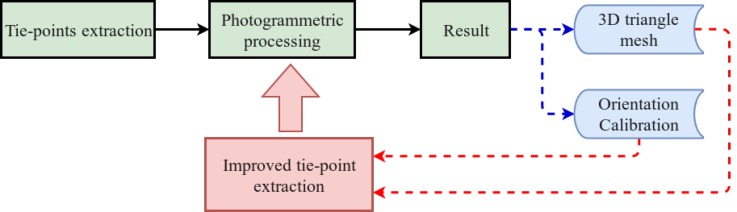
A second iteration processing with refined tie-points.

- the tie-point extraction is free from seed points contrary to [3,28], and produces regularly spaced points, also in areas of low-contrast regions where classical tie-point extractors typically fail to identify salient points;
- contrary to [30], our algorithm is not constrained by the presence of dominant 3D planes in the scene, therefore is not constrained to urban reconstructions;
- our initial structure is represented in form of a triangulated mesh, and the re-projection to images of each mesh entity is rectified to a pre-selected geometry; as the perspective deformations are removed, the algorithm is apt to handle wide baseline acquisitions;

The method can be equally used as an alternative to *Iterative Closest Point* (ICP) algorithm [31] for automated alignment of two individual 3D pointclouds captured from very different viewpoints. See Figure 1 for a summary of the proposed processing. All concieved algorithms were implemented in MicMac—the free, open-source software for photgorammetry [32,33]. This paper is an extended version of our preliminary work published in [34].

## 2. Materials and Methods

### 2.1. An Overview

**Figure 2:**
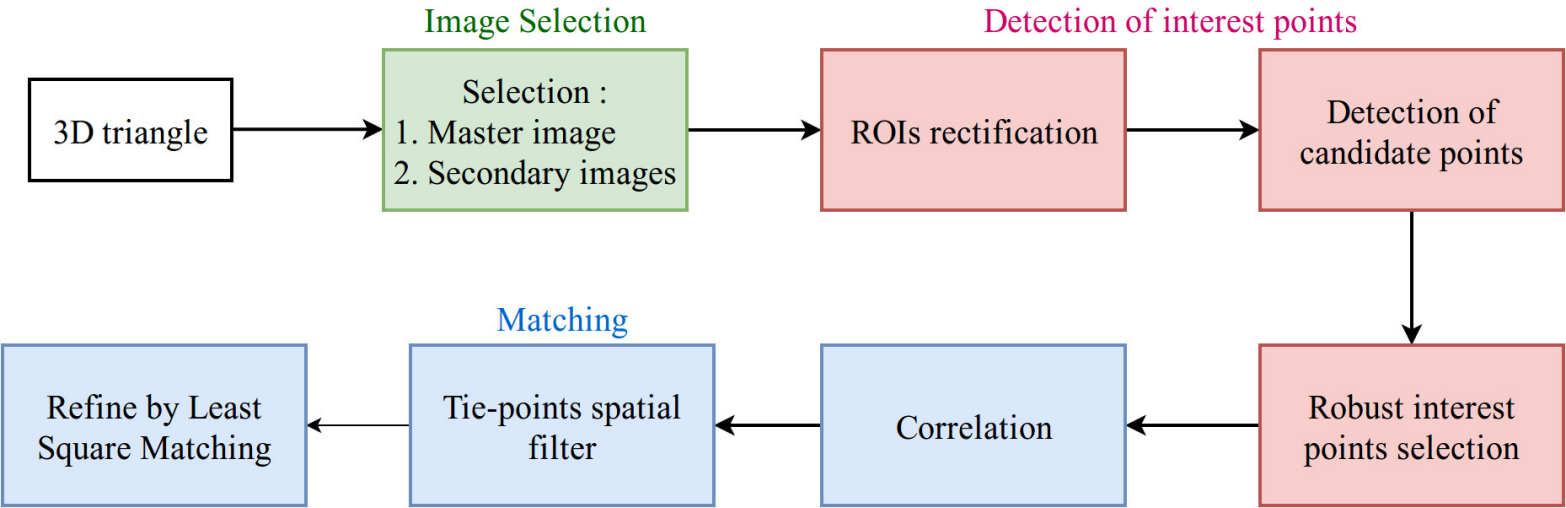
Three general steps of the tie-point extraction method: Image selection, Detection of interest points and Matching.

*SIROP* enhances the obtained result precision by performing the second iteration of photogrammetric processing using the new tie-points (see Figure 1). These new tie-points are extracted based on 3D scene information i.e triangulated mesh. The image orientations and calibrations are used to predict the keypoint matching position and correct the perspective deformation between images before extracting the new tie-points. To assure that the algorithms work properly, it is expected that the approximate (i.e., initial) camera orientation predicts the image points’ positions within 10 pixel precision. *SIROP* method can be described in 3 separate steps as presented in Figure 2:

- Image selection and scene partition.
- Detection of interest points (keypoints).
- Matching by correlation and LSM.

The sequence of steps is applied to each triangle. By iterating over each triangle in the mesh, *SIROP* ensures a homogenous distribution of tie-points in the object space.

### 2.2. Image Selection and Scene Partition

The purpose of this phase is to designate for each triangle a master image and a set of secondary images. The retained images must fulfil two conditions of:

- visibility (here implemented by a Z-buffer filter), and
- minimum resolution (triangles with surface area below 100 pixels will be filtered-out).

**Figure 3:**
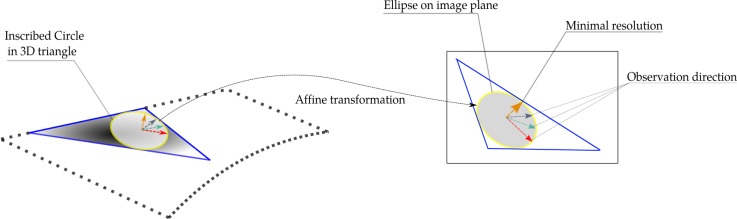
The observation direction and minimal resolution of back-projected triangle.

Out of all images, the master image will be the one with the most optimal resolution. Under perspective deformations, the resolution of a projected surface-element changes according to the observation direction (see minimal resolution direction in Figure 3) within the plane of the 3D triangle. We use here a cautious strategy by defining the minimal resolution obtained among all possible directions, and the master image is the image with the highest minimal resolution value (i.e the optimal resolution). With the optimal resolution one wants to favour a selection of images that are:

- fronto-parallel with respect to the 3D triangle in question, which results in least deformed triangle back-projections, and
- nearer to the triangle in question, which is equivalent of higher resolution.

**Figure 4:**
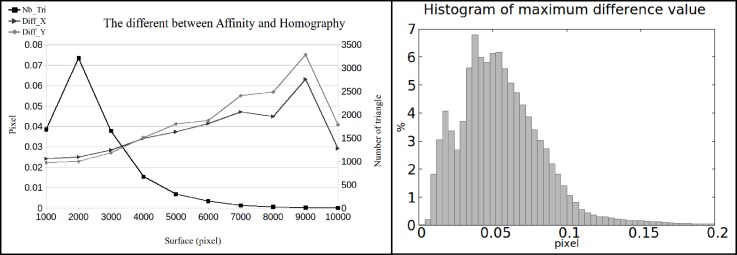
(**Left**): The difference of predicted pixel coordinate between affine and homography transformation in triangle. (**Right**): histogram of maximum difference value in the range of [0,0.2] pixel.

To relate an ellipse defined within the triangle’s plane, and its back-projection in an image, an affine transformation is calculated. The 6 parameters of an affine transformation are estimated from 3 corresponding triangle’s vertices in 3D space and in the image. Rigorously speaking, this plane to plane mapping is described by a homography, but since the mesh elements are small an affine approximation is justified. The errors in image position prediction induced by the adopted approximations are shown in Figure 4. For a triangle of 9000 pixels, the average prediction discrepancy does not exceed 0.08 pixel. The histogram of maximum difference values proves that 99% of discrepancies falls below 0.13 pixel.

**Figure 5:**
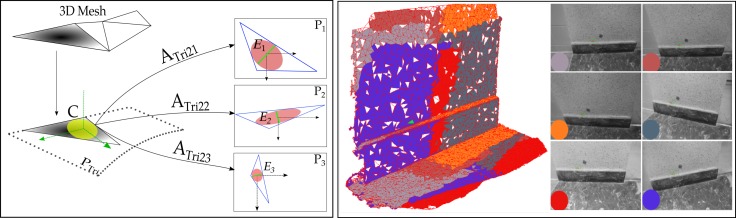
Optimal resolution condition in the selection of master image and scene partition. (**Left**): tensor ellipse in images. $P_{1}$ will be chosen as master image. (**Right**): Scene partition between 6 master images in a dataset. Each color presents a corresponding scene part with each master image.

Mathematically speaking, let $P_{n}$ be the plane of image *n*, $P_{Tri}$ the 3D plane containing the triangle and *C* the circle inscribed in the triangle (see Figure 5). The projection function is approximated by the affine transformation between the 3 vertices of the triangle in its plane in object-space and their back-projections in image *n*. Only $A_{Tri2n}$, the linear part of this transformation, is used for image selection. The 6 parameters are computed from the (2 dimensions) × (3 points) observations with a direct inversion. $E_{n}$ is the ellipse transformed from circle *C* to plane image $P_{n}$ by the affine transformation $A_{Tri2n}$ denoted as follows:

(1) $$A_{Tri2n}=\left[\begin{array}{cc} A_{x_{n}} & B_{x_{n}}\\ A_{y_{n}} & B_{y_{n}} \end{array}\right]=\left[\begin{array}{cc} {\vec{u}}_{n} & {\vec{v}}_{n} \end{array}\right]$$

For each point (cos(θ),sin(θ)) on circle *C*, we have a corresponding point on the ellipse $E_{n}$ mapped by an affine transformation $A_{Tri2n}$:

(2) $$\begin{array}{c} A_{Tri2n}*\left[\begin{array}{c} \cos\theta \\ \sin\theta \end{array}\right]=\left[\begin{array}{cc} {\vec{u}}_{n} & {\vec{v}}_{n} \end{array}\right]\left[\begin{array}{c} \cos\theta \\ \sin\theta \end{array}\right]\\ ={\vec{u}}_{n}\cos\theta +{\vec{v}}_{n}\sin\theta =\vec{V_{n}\left(\theta \right)} \end{array}$$

Value *min*$\left(\vec{V_{n}\left(\theta \right)}\right)$ Equation (3) represents the minimal possible resolution obtained across all directions in image *n* of circle *C*:

(3) $$\min\left({∥\vec{V_{n}\left(\theta \right)}∥}^{2}\right)=\frac{{\vec{u_{n}}}^{2}+{\vec{v_{n}}}^{2}-\sqrt{\left({\vec{u_{n}}}^{2}-{\vec{v_{n}}}^{2}\right)+4{\left(\vec{u}\cdot \vec{v}\right)}^{2}}}{2}$$

The master image is selected as the maximum of all possible minimal resolutions across all images: $Master=\max(\min\left({∥\vec{V_{n}\left(\theta \right)}∥}^{2}\right))$.

A set of secondary images is chosen by applying a threshold on the minimal possible resolution $\min({∥\vec{V_{n}\left(\theta \right)}∥}^{2})$ between each candidate image and the master image. Finally, after the selection of images for all 3D triangles in mesh, the scene has been partitioned between different master images.

#### 2.2.1. Region of Interest Rectification

**Figure 6:**
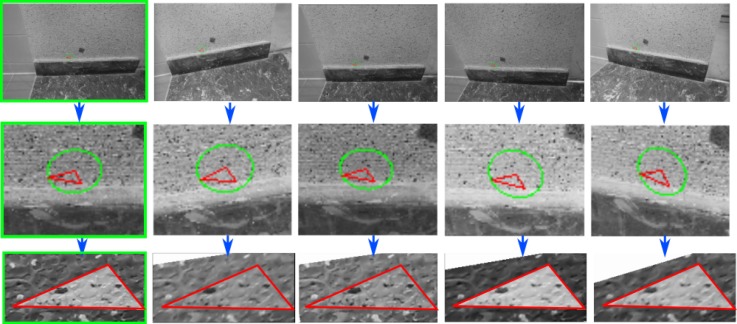
An example of ROI on each images. (**Second line**): triangle ROI projected on images. (**Bottom line**): normalized triangle ROIs. In the green frame is the master image.

For each triangle back-projected to an image, a region of interest (ROI) is defined in its surroundings. All ROIs found in the secondary images are rectified to the geometry of the master image (see Figure 6) using the 6-parameter affine function form master image to the secondary image for this triangle. By doing so, all perspective distorsions due to 3D scene geometry and perspective imaging are removed.

If $\left(x_{s},y_{s}\right)$ is the pixel coordinate in secondary image ROI, the affine mapping is expressed as follows:

(4) $$\left[\begin{array}{c} x_{m}\\ y_{m} \end{array}\right]=\left[\begin{array}{cc} a_{1} & b_{1}\\ a_{2} & b_{2} \end{array}\right]\left[\begin{array}{c} x_{s}\\ y_{s} \end{array}\right]+\left[\begin{array}{c} a_{0}\\ b_{0} \end{array}\right]$$

### 2.3. Detection of Interest Points

This step generates a set of interest points for all back-projected and rectified triangles. In the subsequent steps the found interest points will guide the sub-pixel matching (see Section 2.4).

**Figure 7:**
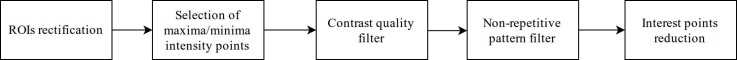
An overview of the interest point detection algorithm.

Firstly, local intensity maxima/minima points are adopted as candidate points. Then, these candidates are filtered by several criteria such as contrast quality, risk of repetitive patterns and spatial distribution. The general workflow is presented in Figure 7, each filter will be detailed in the following section.

#### 2.3.1. Contrast Quality Filter

The purpose of this filter is to eliminate low-contrast candidate points. The idea of this filter is based on the FAST [35] and SUSAN [17] detectors, by examinating the neighboring points to decide if a point is contrast enough. There are three main reasons for this approach selection:

- Fast processing speed: in comparing to Difference of Gaussian approach detector such as SIFT.
- Perform on non-perspective ROI: as presented in Section 2.2.1, perspective in ROI is eliminated. Therefore, a scale rotation invariant approach is unnecessary.
- Interest point localization: interest points are detected at pixel exact and FAST filter-based approach work on pixel exact also. Therefore, we avoid the sub-pixel point localization problem in SIFT approach [36]. Region-based matching method by correlation and Least Square Matching in matching step will localize tie-point at sub-pixel precision.

**Figure 8:**
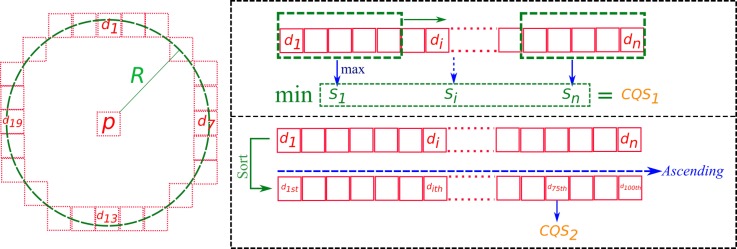
The Contrast Quality Score (CQS). *p* is the intensity value, *d* is the difference in intensity value. $CQS_{1}$ is the contiguous contrast and $CQS_{2}$ is the global contrast score.

The intensity value of a candidate point is denoted as *p*, and we examine its neighboring points (1...n) with intensity values $\left(p_{1}...p_{n}\right)$, respectively. There are a total of 24 neighboring points located on a circle with a radius R=4. A difference in the intensity value $d_{i}$ is computed for each neighboring point at *i* and the central point as $\left|p-p_{i}\right|$ (see Figure 8).

To evaluate the contrast quality of a candidate point, a Contrast Quality Score (CQS) is computed. The CQS is composed of two terms:

- contiguous priority contrast score $CQS_{1}$, and
- global contrast score $CQS_{2}$.

**Figure 9:**
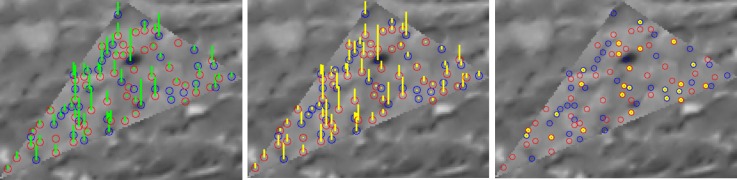
An example of Contrast Quality Score and contrast filter performance.

A candidate point is considered to be of good contrast if at least 75% of differences $d_{i}$ pass an appropriate threshold. The 75th percentile value in the ascending sort difference intensity vector $V_{d}$ is then taken as $CQS_{2}$ score. Then, a sliding window scans through the vector $V_{d}$. Each window score is taken as the maximum value of all elements within the window. $CQS_{1}$ score is the minimum value among the window scores (see Figure 8). Candidate points are considered to be contrasted if $CQS_{1}$ passes a certain threshold. Figure 9 illustrates the performance of the contrast filter on an example.

Finally, a combination of two terms $CQS_{1}$ and $CQS_{2}$ with a weighting factor give a final Contrast Quality Score for each point.

(5) $$CQS=CQS_{1}+2\times CQS_{2}$$

This score will be used in the following interest point reduction step to determine the point’s priority.

The figure below presents an example of Contrast Quality Score for interest point and contrast filter performance. On the left image, interest points are colorized as blue/red circle corresponding to local intensity minima/maxima points. A green bar represents contiguous contrast score term $CQS_{1}$. The middle image represents Global contrast score term $CQS_{2}$ with a yellow bar assigned to each point in triangle. Finally, the right-most image represents points rejected by the filter, which are colorized in yellow.

#### 2.3.2. Non-Repetitive Pattern Filter

**Figure 10:**
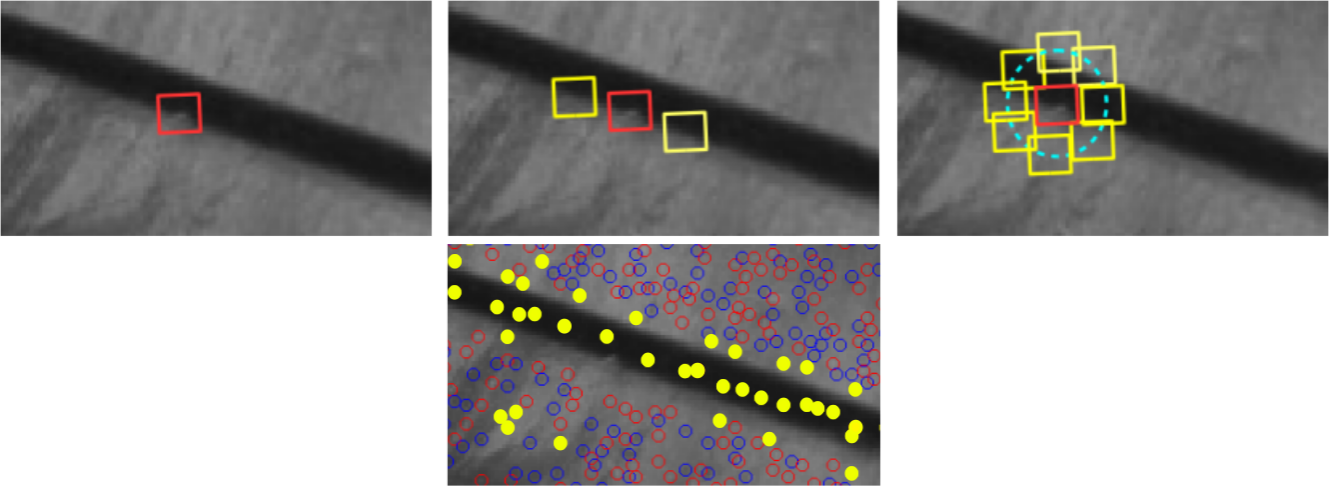
An example of non-repetitive patterns filter.

To make the subsequent matching procedure more robust, candidate points with adjacent repetitive patterns are rejected. To do that, for each candidate point, a circle of neighboring patches around at a certain radius is chosen (see Figure 10). Then, a correlation score is computed between the center patch and each of the neighboring patches. A point is eliminated if the high-score normalized correlation Equation (6) is found along any direction. In this implementation, we chose 0.85 as the high-score value.

Figure 10 shows an example of the performance of this filter. The upper image line presents an ambiguity of interest point located on a linear pattern (red rectangle), which can cause a false-positive in correlation (yellow rectangle). On the bottom image, blue/red circles represent minima/maxima local points, respectively. The yellow points are rejected by the filter because they lie on a linear pattern that may cause an ambiguity in correlation.

#### 2.3.3. Interest Point Reduction

**Figure 11:**
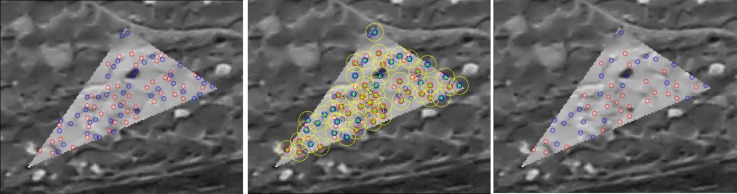
An illustration of the interest point reduction. (**Left**): original points. (**Center**): filter area (yellow). (**Right**): filter result.

After the interest points are selected, a filter is applied to reduce their quantity. The interest points are sorted by CQS to form a processing list, with the priority corresponding to the score value. For each master image, for all interest points, the filter then defines a circular region centered at a point (see Figure 11). Only one point with highest CQS within the circle is retained. The circular region size is chosen relatively to image resolution.

### 2.4. Matching

The interest points in master images must now be assigned their correspondences in the secondary images, i.e., the tie-points are created. Because our image patches are rectified to the geometry of the master image, an area-based matching method can be used to find the correspondences. *SIROP* adopts the normalized cross-correlation technique, followed by least squares matching.

#### 2.4.1. Matching by Correlation

**Figure 12:**
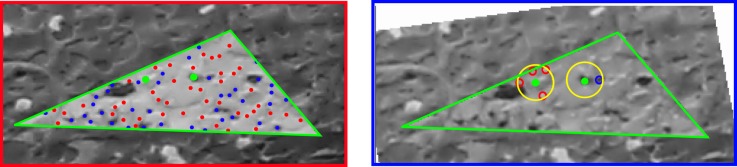
The search region (yellow circle in secondary image ROI-right) and potentially matching points (same characteristic point: max (red), min (blue)) correspond with considered interest point in the master image (left-green). The green triangle frame is ROI normalized.

Zero Mean Normalized Cross-Correlation (ZNCC) (see Equation ( 6)) is adopted to provide the initial tie-point’s position. For each interest point in the master image, ZNCC is computed for all potential interest points in the secondary images. Finally, the point with the highest matching score is selected as the tie-point. In analogy to interest points in the master images, the potential interest points in secondary images are found as maxima/minima within specified circular regions (see the search region in Figure 12).

(6) $$ZNCC_{fg}=\frac{\sum_{i=1}^{N}\sum_{j=1}^{M}\left(f\left(x_{i},y_{j}\right)-\bar{f}\right)*\left(g\left(x_{i},y_{j}\right)-\bar{g}\right)}{\sqrt{\sum_{i=1}^{N}\sum_{j=1}^{M}{\left(f\left(x_{i},y_{j}\right)-\bar{f}\right)}^{2}*\sum_{i=1}^{N}\sum_{j=1}^{M}{\left(g\left(x_{i},y_{j}\right)-\bar{g}\right)}^{2}}}$$

where *f* and *g* as two image patches of size N∗M, $\bar{f}$ and $\bar{g}$ are the corresponding patch average intensity values.

To speed-up the processing, the correlator operates in a multi-scale approach [37]. Three main ZNCC calculation phases can be distinguished:

- at 2-times down-sampled resolution (every other pixel is discarded),
- at the nominal resolution, and
- at sub-pixel resolution (up to 0.1 pixel).

#### 2.4.2. The Tie-Point Spatial Filter

The spatial filter decimates the tie-points previously matched so that their homogeneous distribution in image space is maintained. The following selection criteria are imposed:

- prioritisation of points with high ZNCC scores,
- prioritisation of points with with high manifold,
- even distribution of points in both master and secondary images.

**Figure 13:**
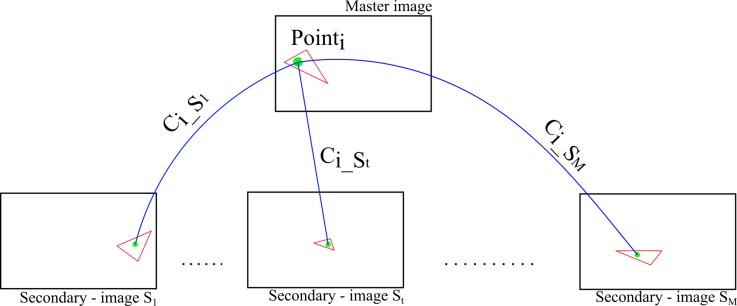
Computation of Global Correlation Score ($C_{i}^{GCS}$) for one tie-points.

Please note that our tie-points are manifold points (i.e., observed on more than two images). To determine which is the better correlated multiple point, for each tie-point in the master image, a Global Correlation Score $C_{i}^{GCS}$ is computed (see Figure 13 and Equation (7)). This score includes all multiple correlation scores between points in the master image and all of its matched points in secondary images.

(7) $$C_{i}^{GCS}=\sum_{t=1}^{M}\left(\frac{1}{\epsilon +(1-C_{i\_S_{t}})}\right)\;\;if\frac{1}{\left(1-C_{i\_S_{t}}\right)}==0\to \epsilon =0.02$$

where $C_{i\_S_{t}}$ as a correlation score Equation (6) for tie-point *i* between master image and secondary image $S_{t}$, with t=[1:M] and *M* is the manifold.

**Figure 14:**
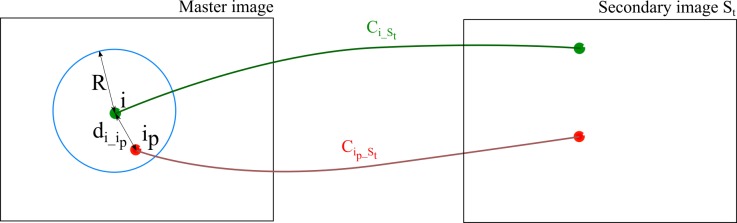
An illustration for terms in Equation (8). $C_{i\_S_{t}}$ is the correlation score between point *i* on master image and its matched point on secondary image $S_{t}$.

**Figure 15:**
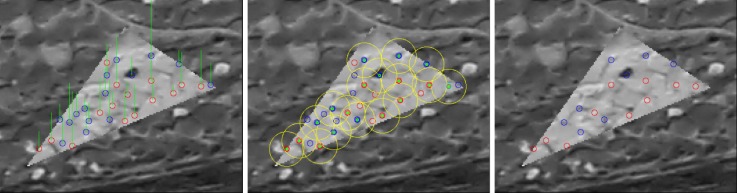
Tie-point spatial filter applied in the priority of points score $C_{i}^{GCS}$: (**Left**): matched points with their score illustrated in green line, (**Middle**): filter space area in the yellow circle, (**Right**): Points after filter.

Equation (7) can be interpreted as a quality indicator composed of the correlation score and the manifold. Points with higher manifold and good correlation will have higher score, hence higher position in filter processing queue. For each current point in queue, neighbor points are picked as all points lying within a radius *R* around it (see blue circle in Figure 14). All points retained from the circle will be tested with Equation (8) which is a function of a correlation score between two potentially corresponding points (i.e., $C_{i\_S_{t}}$ calculated with Equation (6)) and the normalized distance $\frac{d_{i\_i_{p}}}{R}$ between the neighbor $i_{p}$ and the current point *i*. In other words, points that are close to point *i* and with poor correlation scores will more likely be filtered out. Figure 15 visualizes intermediary results of the described approach. Pseudocode of tie-points spatial filter is described in Algorithm 1.

(8) $$C_{i_{p}\_S_{t}}<C_{i\_S_{t}}+\left(1-{\left(\frac{d_{i\_i_{p}}}{R}\right)}^{2}\right)*0.2$$

**Algorithm 1:** Tie-point spatial filter

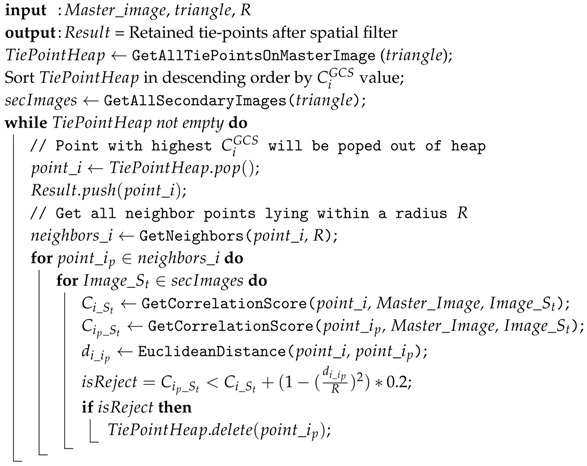



#### 2.4.3. Matching Refinement by Least Square Matching

**Figure 16:**
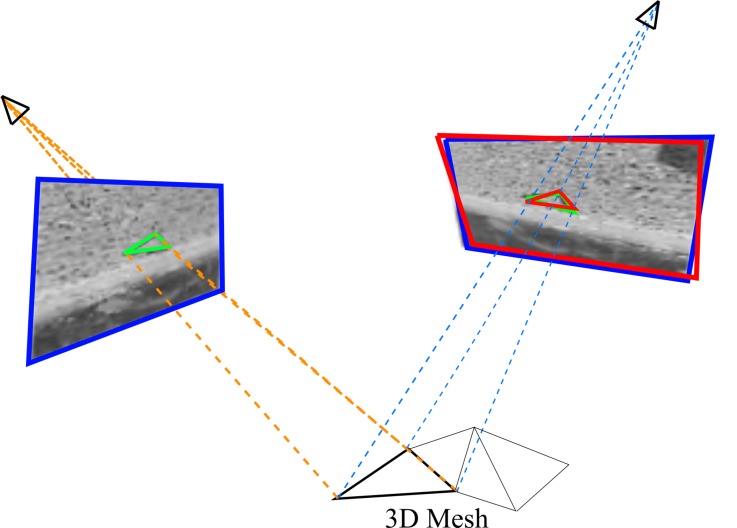
Minor error in image orientation may cause errors in ROI normalization. (**Left**): master image, (**Right**): the blue frame is an image orientation with a minor parallax error, the red one is a corrected orientation. If we normalize the geometry of the ROI in the right image (green triangle region) with respect to the left image by an affine transformation estimated from triangle vertices, they are not exactly in the same geometry.

In the final step, LSM [28] is used in combination with *SIROP* to compensate for unmodelled image orientation errors (i.e., y-parallax), see Figure 16.

Pixel intensity values in image patches are used as observations. Both the affine transformation (6 parameters as previously discussed) and the radiometric model (2 parameters including the contrast and brightness) are the unknowns in the least square adjustment model:

(9) $$\begin{array}{c} g_{1}\left(x_{1},y_{1}\right)=A*g_{2}\left(a_{0}+a_{1}x_{1}+a_{2}y_{1},b_{0}+b_{1}x_{1}+b_{2}y_{1}\right)+B \end{array}$$

where $g_{1}$, $g_{2}$ as two same size image patches of a tie-point given by correlation. A linear model with the contrast (named *A*) and the brightness (named *B*) is adopted to model the intensity variation between the two patches.

To transfer the non-linear model in Equation (9) to a linear one, Taylor linearisation is adopted. All parameters are estimated across several iterations. The stop condition is a pre-defined maximum number of iterations or convergence error estimation between two consecutive iterations.

**Figure 17:**
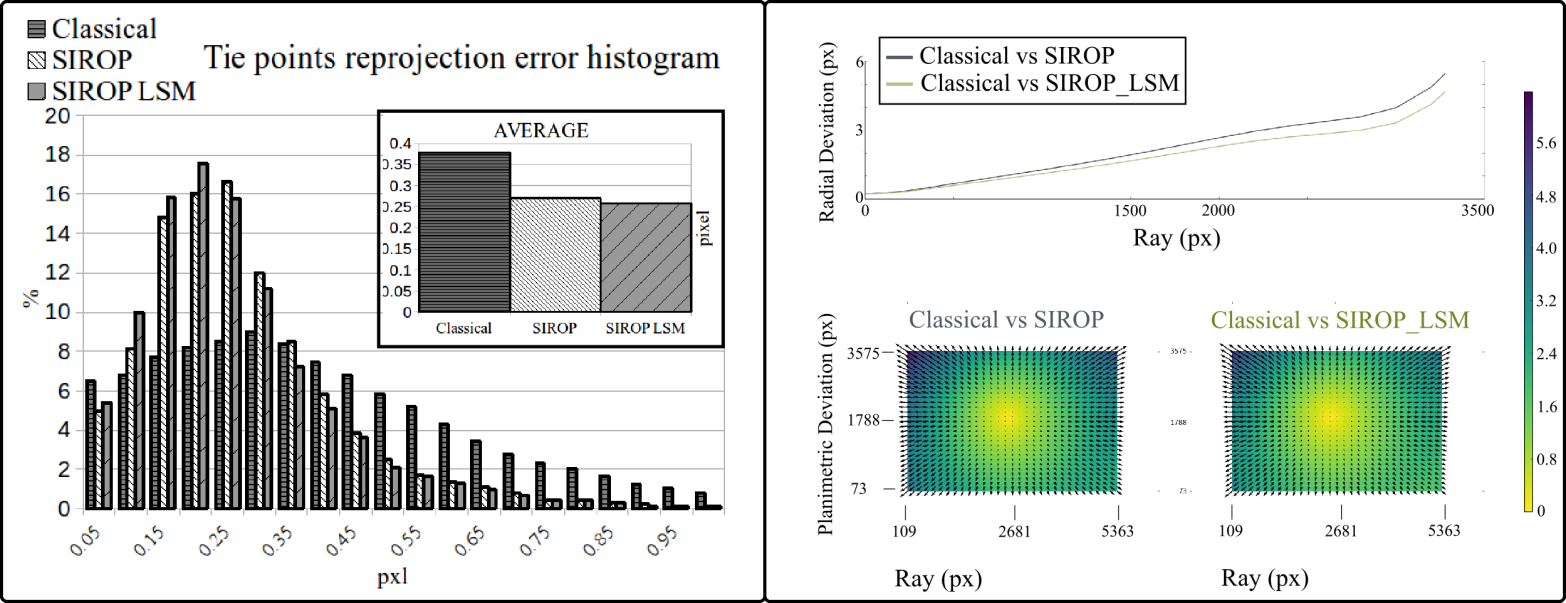
The evaluation of LSM efficient on *SIROP* tie-point. (**Left**): reprojection error histogram. (**Right**): difference in camera calibration with LSM refinement.

To evaluate the gain of employing the LSM, an experiment involving 5 images was performed. Figure 17 shows a bundle block adjustment (BBA) reprojection error improvement of 0.02 pixel, and a minor change in the estimated camera parameters. Given a typical acquisition with thousands of tie-points, the additional LSM processing times should not be neglected. Accordingly, in the current *MicMac* implementation, the LSM is an optional parameter, and the parameter was inactive in all the experiments presented in the next section. LSM refinement is said to improve the *SIROP*’s tie-points precision but it has a drawback of longer computation times.

## 3. Experiments and Results

### 3.1. The Evaluation Procedure

The evaluation is carried out on four datasets with different 3D scene types, cameras and acquisition geometries. To obtain 3D accuracy measures, several Ground Control Points (GCPs) were measured in each scene. Each dataset is processed with two methods: the classical processing with SIFT tie-points (implementation of [38]) (*Classical*) and the *SIROP* method, which only uses its own-detected points. In any case, grayscale images are used to extract tie-points.

The difference between the two processing methods is in the employed tie-points. In both scenarios the camera parameters are estimated in a self-calibration BBA (i.e., the Fraser camera model [39]). The BBA computation is first carried out in a relative coordinate system, and further transferred to the coordinate system of the GCPs with the 7-parameter transformation estimated from all GCPs. Three case studies are summarized in Table 1.

Each case study was processed according to the workflow presented in Figure 1. Due to repetitivity of the obtained results, only one detailed outcome analysis is presented—the terrestrial *Hallway* dataset in Section 3.2. As for the UAV datasets (*Viabon1*, *Corridor*), a compiled presentation of the obtained results is given in Section 3.3.

The evaluation is carried out by looking at the following accuracy measures:

- euclidean distance between GCPs resulting from image triangulation and their true values (*d*).
- the BBA standard deviation.
- color-coded error map of tie-points residuals.

Additionally, histograms of tie-point residuals and their manifold are reported.

### 3.2. The Hallway Dataset

**Figure 18:**
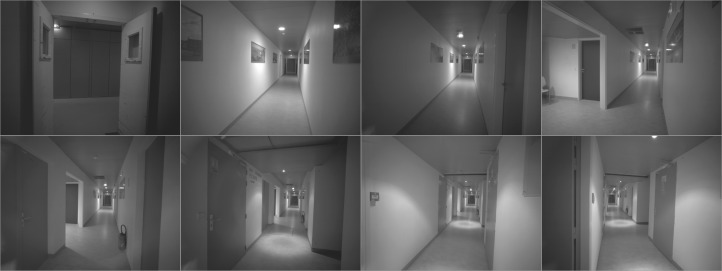
Some images from the *Hallway* dataset. Photogrammetric ultra light Camera IGN CamLight [40], 18 mm focal length.

**Figure 19:**
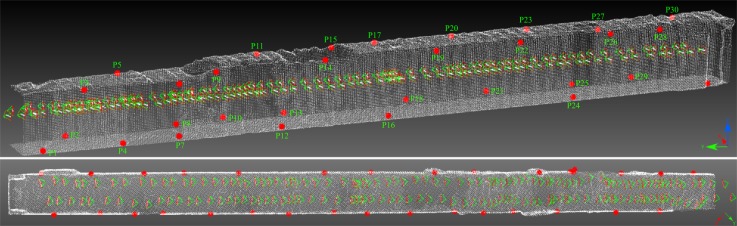
The *Hallway* dataset acquisition with GCP points (red).

**Figure 20:**
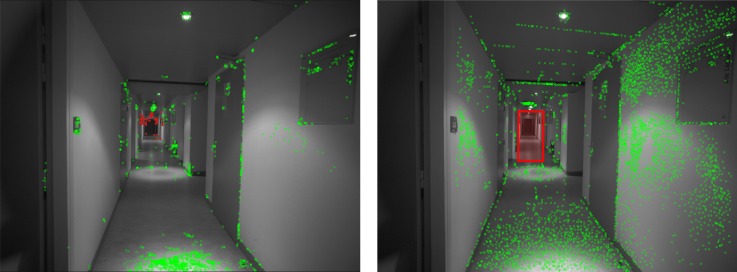
Comparison of the tie-points distribution. These are tie-points between one image with all others images in the dataset. (**Left**): Classical method by SIFT, (**Right**): *SIROP*. Tie-points distribution with *SIROP* is better. Tie-points in the “far” scene part (the red frame in right image and the red points in left image) with low ground resolution is ignored by *SIROP*.

**Figure 21:**
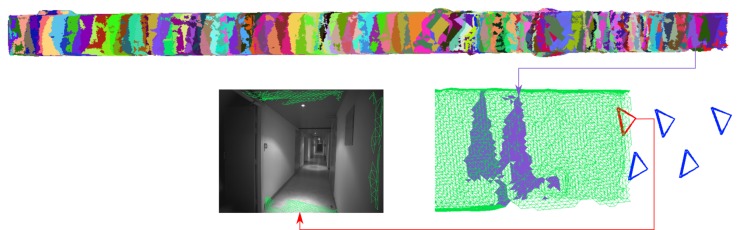
The scene partition and optimal resolution scene part chosen for one master image. (**Top row**): Scene partition. Each color presents the part for one master image in the dataset. (**Bottom row**): (**Right**): Demonstration for one master image (red) with its scene part and secondary images (blue). (**Left**): The scene part on master image.

A snapshot of the dataset and the distribution of GCPs are shown in Figure 18 and Figure 19, respectively. In Figure 20 one clearly sees the advantage of the proposed methodology. The extracted tie-points are, as expected, well distributed in both object and image spaces. The optimal resolution criteria for master image selection, according to which a triangle is assigned its master favouring large image scale, is also kept as revealed in Figure 21.

**Figure 22:**
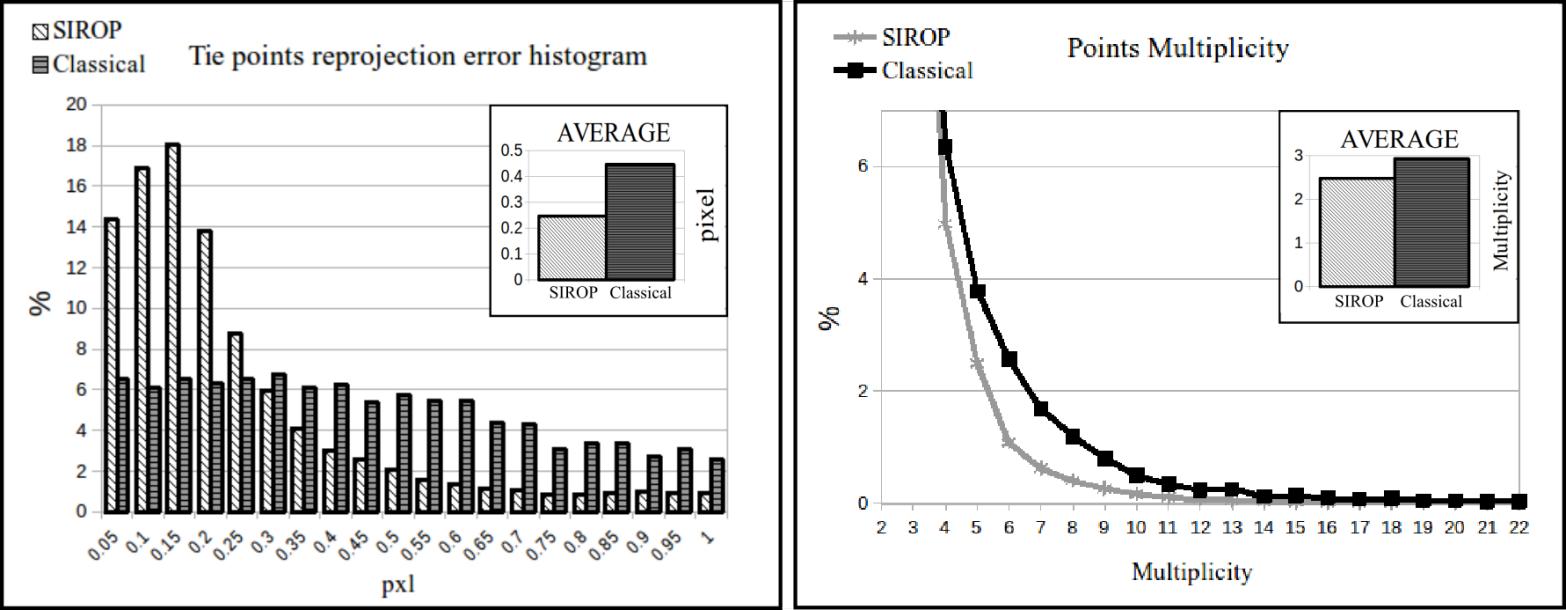
Comparison of the statistics of tie-points properties between two processing methods. *SIROP* produces tie-points with smaller re-projection error but less multiplicity in this specific case. (**Left**): re-projection error histogram. (**Right**): Multiplicity.

**Figure 23:**
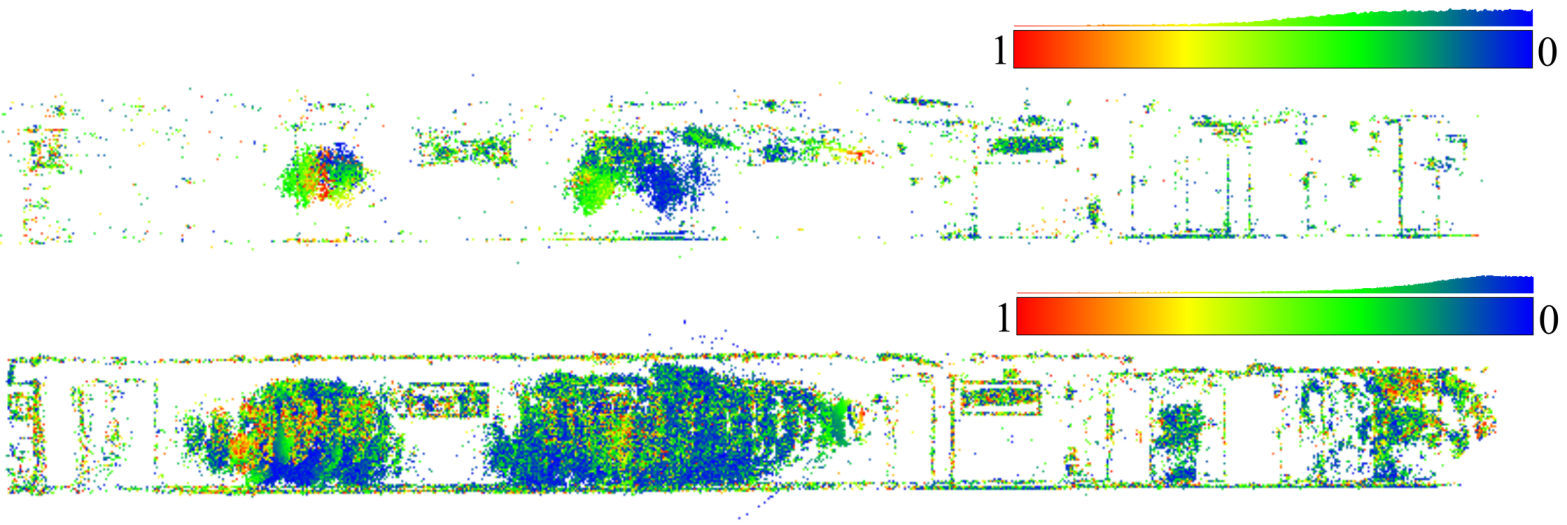
Tie-points in 3D space with its re-projection error coded in color. The color scale bar presents a histogram of re-projection error in range [0,1] pixel. (**Top**): classical processing with SIFT, (**Bottom**): by *SIROP*. The holes in the model caused by very low dynamic image in these scene parts.

**Figure 24:**
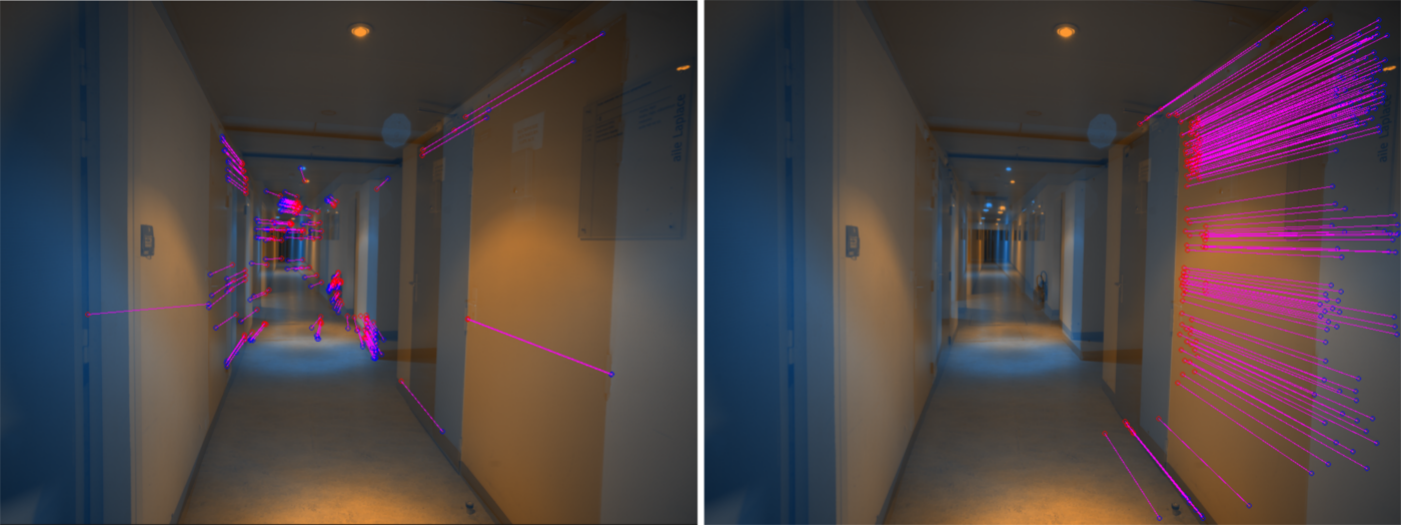
An explanation for the reduction of tie-points multiplicity by *SIROP* in *Hallway* dataset. These are tie-points in one image couple in the dataset. (**Left**): SIFT detects tie-point at the far end of the scene, so these tie-points have a high multiplicity because it appears on almost all image couples. (**Right**): our method extracts tie-points in the optimal resolution of scene, so all the “far” scene part is eliminated.

**Figure 25:**
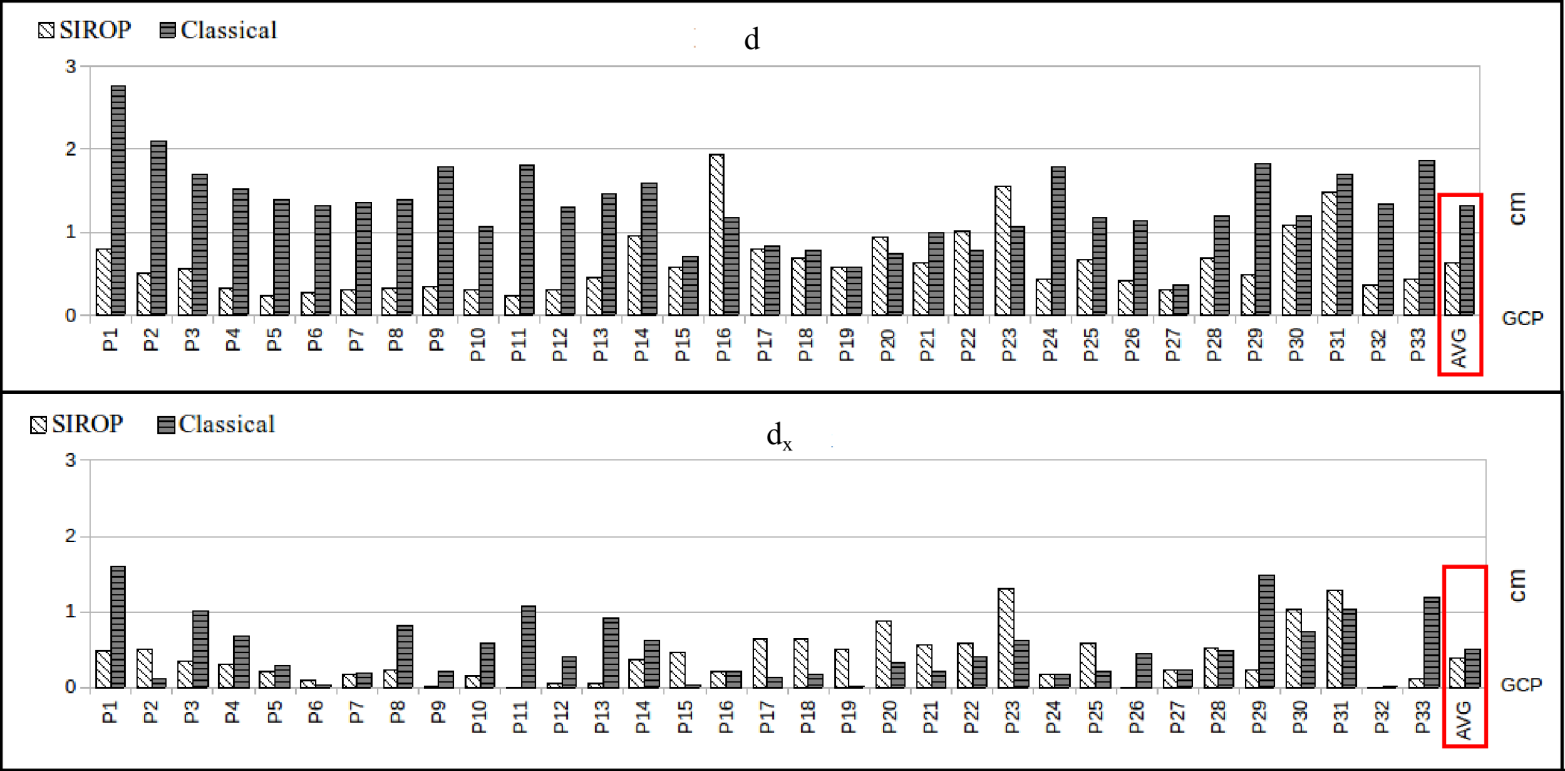

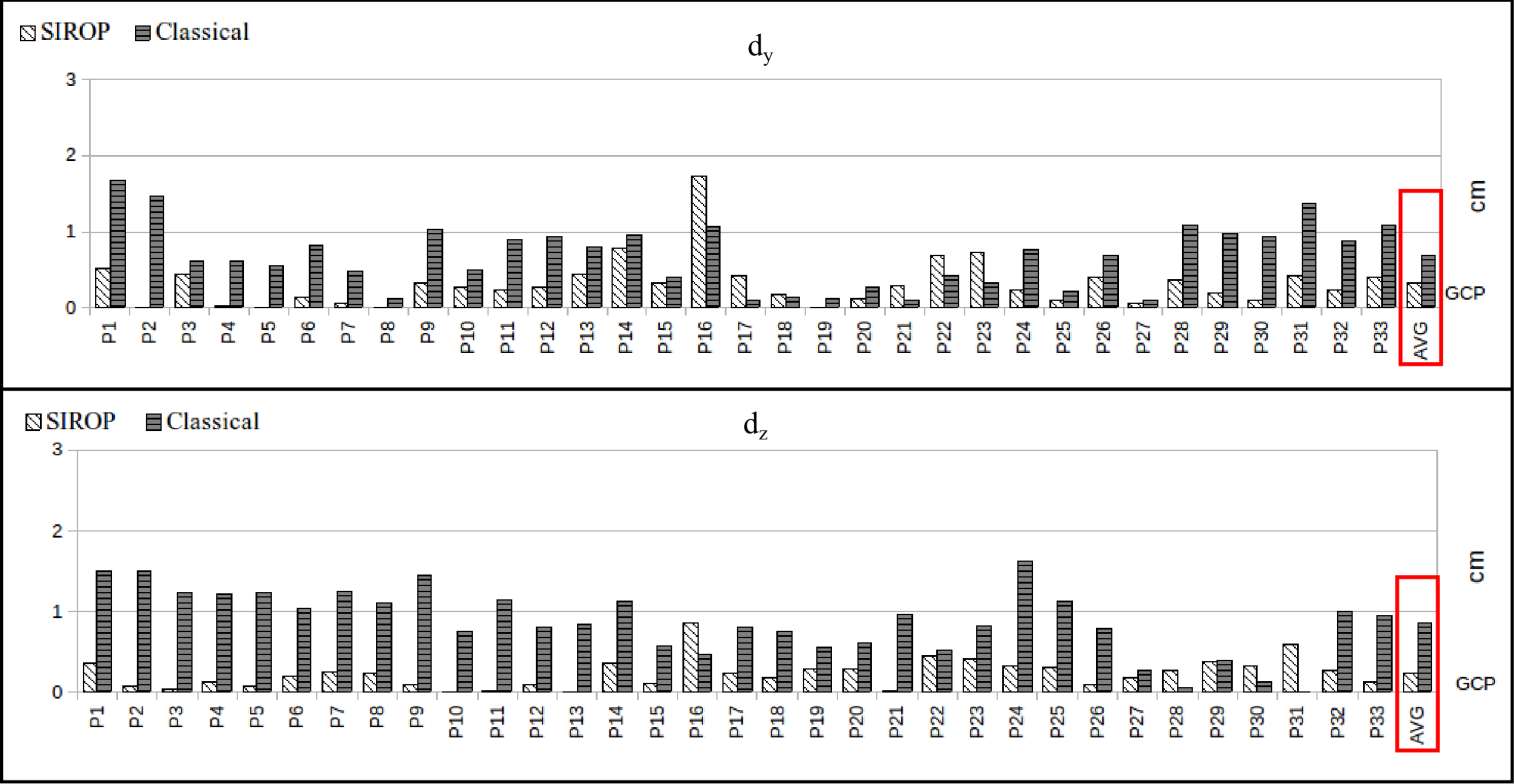
Comparison with GCPs—the *Hallway* dataset. From the top: Euclidean distance, in X, in Y, in Z. In the red frame: average distance value.

*SIROP* reduces the tie-point residuals (see Figure 22 and Figure 23) on average by almost a factor of two. Tie-points manifold is slightly less favourable which is due to the specificity of the long-corridor acquisitions. Throughout the acquisition the camera points at infinity whereas *SIROP* privileges points close to the camera. As a result, the far points are ignored (see Figure 24 for evidence). Finally, Figure 25 reports on distances between the true value and the photogrammetrically-predicted positions of all GCPs in the *Classical* and the *SIROP* processing scenarios. An average the distances decreased by a ratio of 1.2, 2.1 and 3.7 respectively in *X*, *Y* and *Z*, which is equivalent of $1.4\sigma_{x}$, $3.6\sigma_{y}$, $15.8\sigma_{z}$.

### 3.3. UAV Datasets

**Figure 26:**
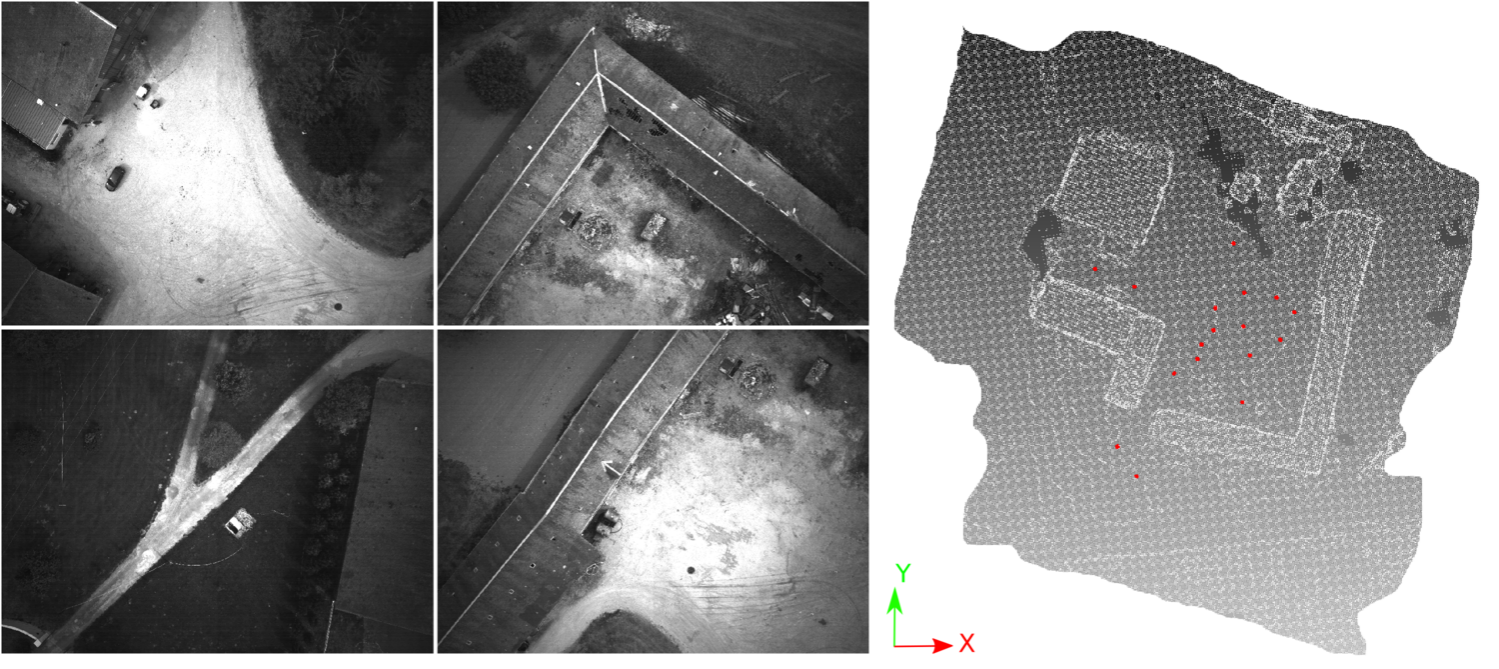
The Viabon 1 dataset.

The aim of this section is to asses the performance of *SIROP* in an aerial acquisition. Two UAV-acquisitions were processed: a block acquisitions (*Viabon1*—see Figure 26), and a corridor acquisition (*Corridor*).

As detailed in Table 2, *SIROP* outruns the *Classical* method in all the tested scenarios. The accuracy gain is observed particularly along the Z component, i.e., along the coordinate with the highest estimation uncertainty due to the imaging configuration. Global increase in tie-points manifold is also observed.

**Figure 27:**
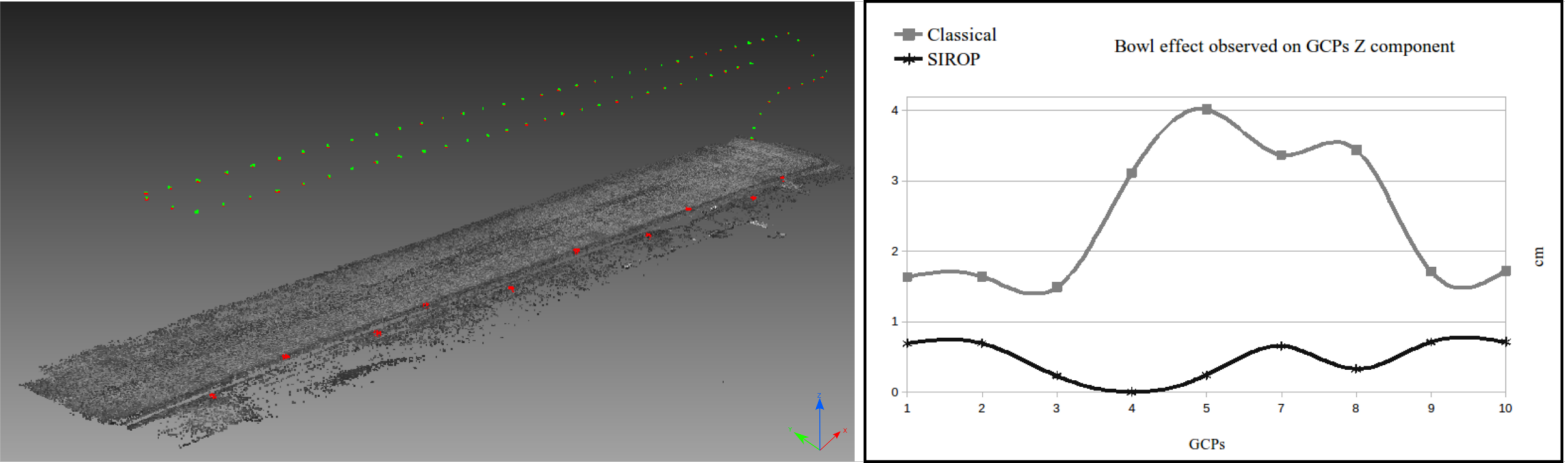
The non-linear distortion effect correction on *Corridor* dataset. (**Left**): Linear acquisition protocol and GCPs distribution (red point). (**Right**): comparison of 3D uncertainty on Z shows the dome effect with classical processing, and the correction obtained by *SIROP*.

**Figure 28:**
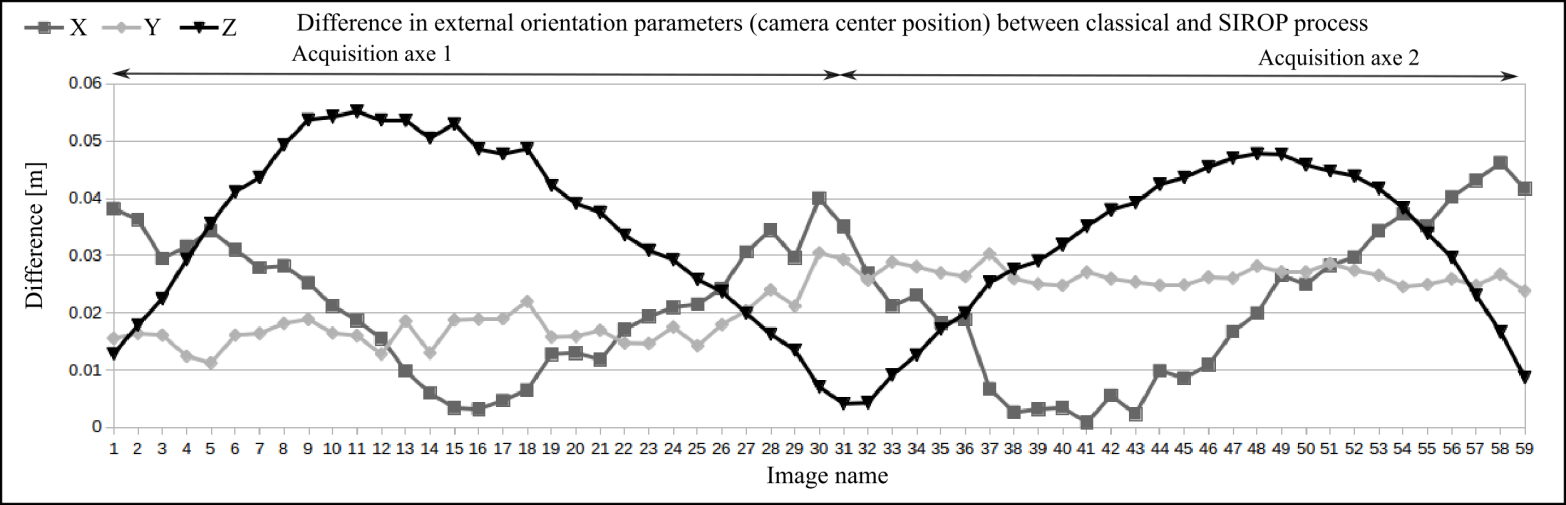
The difference of camera center positions in X,Y,Z of *Corridor* dataset after SIROP processing.

The *Corridor* dataset uncovers another beneficial aspect of *SIROP*, i.e., the capability of suppressing the “dome” effect. It has been stated by many researchers [8,41,42] that under certain imaging configurations the self-calibrating BBA is suboptimal. The arising correlations between the camera internal parameters and the exterior orientations lead to incorrect modelling of both. As a result, one observes the so-called “dome” effect in the reconstructed pointcloud. One way to overcome is to employ a denser network of GCPs or include oblique views. However, it is not always possible. In such circumstances, *SIROP* proves that higher accuracy and well distributed tie-points can equally mitigate the effect (see Figure 27 and Figure 28).

**Figure 29:**
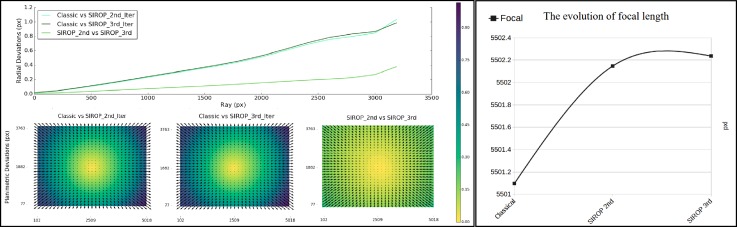
The evolution of internal camera parameters in *Corridor* after second and third *SIROP* iteration. (**Left**): The radial distorsion. (**Right**): The camera focal length.

The study of the evolution of the camera’s internal parameters confirms that with the *SIROP*’s tie-points one is able to model the camera’s systematic errors more efficiently. Please note the difference in residuals at the margins of the image for the *Classical* and *SIROP* processing, as is presented in Figure 29. Stability of the estimated parameters is also tested by running a second and third *SIROP* iteration.

## 4. Conclusions and Discussion

In this article, a novel photogrammetric processing chain called *SIROP* and aimed at enhanced precision 3D reconstruction is proposed. The algorithms make use of the approximate 3D scene geometry and the known camera orientations to generate a new set of optimal tie-points. With the new tie-points a more precise camera parameters and orientation are estimated, and therefore more accurate 3D measurements can be done. The methodology is implemented in the free open-source software for photogrammetry—MicMac.

**Figure 30:**
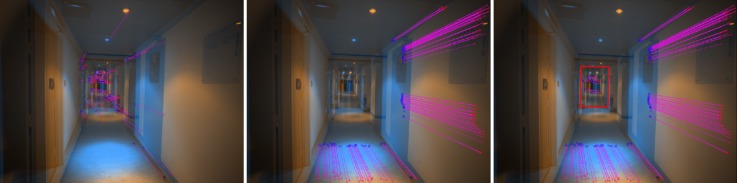
Completion of tie-point extractions by SIFT in low resolution scene part and *SIROP* in optimal resolution part. (**Left**): tie-points by SIFT. Middle: tie-points by *SIROP*. (**Right**): SIROP and SIFT points are taken to complete the “far” low resolution scene part (red frame).

The performance of the introduced algorithms was assessed in three different scenarios: an indoor acquisition (*Hallway*), and two UAV acquisitions (*Viabon1*, *Corridor*). The outcome accuracy were investigated on several GCPs, giving an improvement by a ratio of 2.09, 2.83, 1.46 for the respective datasets. The tie-points manifold was increased in all but the *Hallway* dataset where far points were eliminated contributing to a slightly lower points’ redundancy. To alleviate the issue and the potential camera’s rotational drift, the low-resolution tie-points from the *Classical* method could be integrated with the *SIROP* tie-points (see Figure 30).

Inspection of the UAV datsets revealed that *SIROP* tie-points contribute to a better estimation of the camera internal parameters and thus are capable of eliminating the systematic “dome” effect. Stability of the internal camera parameters calculated with *SIROP* has been also verified (see Figure 29).

The processing time of SIROP is also evaluated by the *Corridor* dataset (see Figure 27 and Table 2) containing 77 panchromatic 20-megapixel images. The classical framework at full-resolution, from the tie-points extraction to 3D mesh generator requires 234 minutes. SIROP is then applied using the 3D mesh and image orientation derived from the classical round. SIROP time consuming essentially depends on the number of images in dataset and the number of triangles in the 3D mesh. In this test, the 3D mesh contains 18021 3D triangles and SIROP processing takes 18 minutes.

Overall, the method proved its efficiency and could be adopted as a post-processing step for fine metrology applications. Furthermore, *SIROP* could be used to accelerate the classical processing by using a very low resolution scene geometry calculated with a SIFT-like method at its input.

## Figures and Tables

**Table 1:** Description of four datasets used to evaluate the method.

Dataset Name	*Hallway*	*Viabon 1*	*Corridor*
*Type*	Terrestrial/Indoor	Drone/Outdoor	Drone/Outdoor
*Camera*	IGN Camlight	IGN Camlight	IGN Camlight
*Focal Length*	18 mm	35 mm	35 mm
*Images*	179	118	77
*Scene Size* • (L∗W)m	30 × 1.5	140 × 140	437 × 65
*Nb GCP* • Type • Incertitude	33 Tophography $\sigma_{XYZ}=\left[0.8,1,0.4\right]$ mm	7 GPS RTK $\sigma_{XYZ}=\left[1,1,2\right]$ cm	10 GPS RTK $\sigma_{XYZ}=\left[1,1,2\right]$ cm

**Table 2:** Comparison between two methods on two datasets: euclidean distance on GCPs, average tie-point reprojection error and manifold.

Datatset	Method	Value					
		d [cm]	d_x [cm]	d_y [cm]	d_z [cm]	Rep_Err [pxl]	Multiplicity [Points]
Viabon 1	Classical	3.43	0.71	0.71	3.17	0.59	5.2
	SIROP	1.21	0.64	0.69	0.52	0.31	5.6
Corridor	Classical	2.13	0.86	0.8	1.44	0.29	3.2
	SIROP	1.45	0.66	0.75	0.82	0.13	4.2

## Abbreviations

The following abbreviations are used in this manuscript:

SIROP	Second Iteration to Refine image Orientation with improved Tie-Point
ICP	Iterative Closest Point
LSM	Least Square Matching
ROI	Region of interest
CQS	Contrast Quality Score
ZNCC	Zero Mean Normalized Cross-Correlation
BBA	Bundle Block Adjustment
GCP	Ground Control Point
UAV	Unmanned aerial vehicle
